# Are We Monitoring Our Lithium Patients as Well as We Should be? A Two-Cycle Audit Evaluating Lithium Clinic Documentation in a District General Hospital in Wales

**DOI:** 10.1192/bjo.2024.604

**Published:** 2024-08-01

**Authors:** Huda Mohammed, Rahatul Islam, Cressida Sparrow, Timothy Chan

**Affiliations:** Princess of Wales Hospital, Bridgend, United Kingdom

## Abstract

**Aims:**

Lithium is a mood-stabiliser with a narrow therapeutic index. Patients are known to be at risk of lithium toxicity if they are unaware of how to recognise its signs. NICE guidelines outline the information that must be relayed to these patients. Furthermore, *GMC Good Medical Practice* highlights the importance of clear and contemporaneous patient records that contain relevant clinical information.

The aim of this two-cycle audit was to assess the quality of documentation for patients reviewed in lithium clinic and to analyse the consistency of the notes recorded to ensure high quality care provision and communication within the department, in line with the NICE guidelines.

**Methods:**

The inclusion criteria were patients over the age of 65, prescribed lithium and were actively reviewed in the monthly lithium clinic at the Older Person's Mental Health Service (OPMHS) at Princess of Wales Hospital in Wales.

A data collection form was created to ensure all the relevant data in line with NICE guidelines was captured including serum lithium level, lithium dose, other psychotropics, side effects, renal function, patient mood, safety netting advice provided including signs of toxicity and awareness of lithium card. A standard of 100% was set for this data to be captured for each patient.

**Results:**

Cycle 1 was completed in November 2023 where a total of 18 patient records were selected (N = 18). Lithium dose, lithium level and renal function were recorded in over 83.3% (n = 15) of the files audited. Details on psychotropics were recorded in 61.1% (n = 11), side effects in 50% (n = 9) and patient mood in 77.8% (n = 14). Safety netting advice was recorded in 11.1% (n = 2). Furthermore, it was noted data recorded varied between clinicians.

The results of this audit were disseminated to OPMHS team. A proforma was introduced to encourage capture of all relevant information and to ensure consistency. Feedback was collected from clinicians using the proformas and relevant changes were made.

A second cycle of this audit was carried out after the proforma was introduced to the subsequent clinic (N = 12). This showed an improvement in record-keeping including lithium dose, lithium levels, psychotropics and side effects of 100% (n = 12). Renal function and mood were recorded in 91.7% (n = 11) of files and safety netting advice provision in 75% (n = 9) of files audited.

**Conclusion:**

Introduction of a proforma is a simple and effective way to ensure relevant and important details are documented. This is not only for good clinical practice, but for medico-legal reasons also.

